# 28-Hydroxy-3-oxoolean-12-en-29-oic Acid, a Triterpene Acid from *Celastrus Orbiculatus* Extract, Inhibits the Migration and Invasion of Human Gastric Cancer Cells In Vitro

**DOI:** 10.3390/molecules24193513

**Published:** 2019-09-27

**Authors:** Zewen Chu, Haibo Wang, Tengyang Ni, Li Tao, Liangliang Xiang, Zhen Zhou, Yayun Qian, Masataka Sunagawa, Yanqing Liu

**Affiliations:** 1 Institute of Translational Medicine, Medical College, Yangzhou University, Yangzhou 225001, China; 2 The Key Laboratory of Syndrome Differentiation and Treatment of Gastric Cancer of the State Administration of Traditional Chinese Medicine, Yangzhou 225001, China; 3 The Key of Cancer Prevention and Treatment of Yangzhou University, Yangzhou 225001, China; 4 Department of physiology, School of Medicine, Showa University, Tokyo 142, Japan

**Keywords:** *Celastrus orbiculatus*, EMT, invasion, migration, MMP

## Abstract

Gastric cancer is the fifth most common tumor and has the third-highest mortality rate among various malignant tumors, and the survival rate of patients is low. *Celastrus orbiculatus* extract has been shown to inhibit the activity of a variety of tumors. This study explored the inhibitory effect of the oleanane-type triterpenoid acid 28-hydroxy-3-oxoolean-12-en-29-oic acid molecule from *Celastrus orbiculatus* extract on gastric cancer cell invasion and metastasis and determined its mechanism. 28-Hydroxy-3-oxoolean-12-en-29-oic acid was first diluted to various concentrations and then used to treat SGC-7901 and BGC-823 cells. Cell proliferation was assessed by an MTT (thiazole blue) assay. Transwell and wound healing assays were used to assess cell invasion and migration. High-content imaging technology was used to further observe the effects of the drug on cell invasion and migration. Western blotting was used to assess the effects on the expression of matrix metalloproteinases (MMPs) and the effects on epithelial–mesenchymal transition (EMT)-related proteins and phosphorylation-related proteins. We found that 28-Hydroxy-3-oxoolean-12-en-29-oic acid inhibited the migration and invasion of SGC-7901 and BGC-823 gastric cancer cells in a dose-dependent manner. Consequently, 28-hydroxy-3-oxoolean-12-en-29-oic acid decreased the expression of EMT-related proteins and MMPs in gastric cancer cells and reduced protein phosphorylation, inhibiting the migration and invasion of gastric cancer cells.

## 1. Introduction

Gastric cancer (GC) is one of the most common malignancies in the world, with mortality worldwide. In China, the incidence rate of gastric cancer in 2015 was 15.8%, ranking second among cancers, and the mortality rate was 17.6% [1]. Patients with early gastric cancer have a good prognosis, and the curative effect of radical surgery is significant. The 5-year survival rate is 85–90% [2], but the early detection rate of gastric cancer is low. More than 75% of Chinese gastric cancer patients are diagnosed after the disease has progressed to the middle and late stages. Currently, there are few clinical treatment methods for gastric cancer. The overall survival rate of patients treated with surgery alone is only approximately 19%, and the curative effect is poor [3]. In addition, radiotherapy and chemotherapy are important means for treating malignant tumors, but their side effects are considerable, and they are often used as adjuvant or palliative treatments before or after surgery.

Tumor invasion and metastasis are biological processes regulated by many factors and multiple signaling pathways. Many signaling pathways, such as the Wnt, PI3K/AKT, TGF-β, and Notch signaling pathways, participate in the regulation of these processes and play an important role in tumor invasion and metastasis [4]. Early invasion and metastasis are important causes of death in gastric cancer patients, and the mechanism of tumor invasion and metastasis is very complicated. These processes mainly involve epithelial–mesenchymal transition (EMT), CAM secretion, extracellular matrix (ECM) lysis, tumor cell invasion, mesenchymal-epithelial transformation and tumor angiogenesis [5], and each step is regulated by multiple factors. Continued understanding of the occurrence and mechanism of tumor invasion and metastasis will definitely play an extensive role in the search for and development of effective antitumor drugs and will provide new ideas and approaches for the prevention of cancer.

The rattan shrub *Celastrus orbiculatus* Thunb., which belongs to the genus *Celastrus*, is mainly distributed in Northeast China and Southwest China [6]. *Celastrus orbiculatus* Thunb. was first recorded as a traditional Chinese medicine during the Qing Dynasty in “Zhiwu Mingshi Tukao”—the drug is mild; the cane, roots, leaves, fruits, and seeds of the plant can be used as medicine; and it has effects on hurricane dehumidification, qi and blood circulation, swelling and pain relief, and detoxification [7]. From the root bark, stems, leaves, seeds and other parts, various components with antitumor, anti-inflammatory, antiviral and other activities can be separated, mainly phenylpropanoids, diterpenoids, triterpenoids, etc. [8,9]. In recent years, related experimental studies have found that products from *Celastrus orbiculatus* Thunb. can inhibit a variety of malignant tumors, inhibit tumor cell proliferation, induce apoptosis and autophagy, inhibit angiogenesis, and inhibit tumor cell invasion and migration ability [10,11,12]. Based on this research, the laboratory has successfully separated the polysaccharides from *Celastrus orbiculatus* Thunb. by improving the extraction process and carried out drug sensitivity experiments on the newly separated components. The *Celastrus orbiculatus* oleanane-type triterpenoid acid 28-hydroxy-3-oxoolean-12-en-29-oic acid is the most sensitive, so this component was selected for further study.

In this study, we investigated by in vitro experiments whether the olean-type triterpenoids from *Celastrus orbiculatus* extract inhibit the invasion and metastasis of gastric cancer cells and whether the individual components of *Celastrus orbiculatus* extract have the same anti-invasive effect on the invasion and metastasis of gastric cancer cells in order to clarify the multitargeted and multilinked molecular mechanism. This study provides a more solid theoretical and experimental basis for further development of *Celastrus orbiculatus* Thunb. extract components as antitumor drugs.

## 2. Results

### 2.1. Effect of Oleanane-Type Triterpenoid Acid from Celastrus Orbiculatus Thunb. on the Viability of Human Gastric Cancer Cells

**Figure 1:**
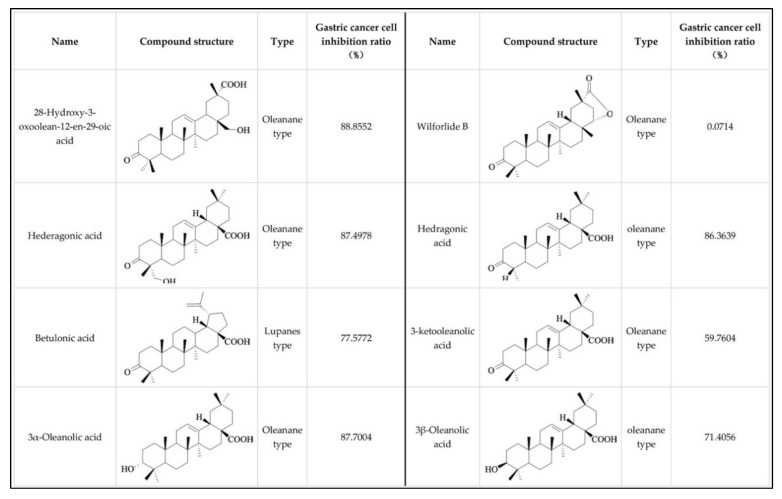
The molecular structure of several oleanane-type triterpenoid acid extracted from the *Celastrus orbiculatus* Thunb. Their names and types, and their inhibition rate to gastric cancer cells are shown in the figure.

We isolated the following oleanane-type triterpenoid acid from the *Celastrus orbiculatus* Thunb, then we used MTT (thiazole blue) assay to detect the inhibition rate of gastric cancer cells one by one (Figure 1). According to the results, the 28-hydroxy-3-oxoolean-12-en-29-oic acid is the most sensitive, so this component was selected for further study.

### 2.2. Effect of 28-Hydroxy-3-oxoolean-12-en-29-oic Acid on the Viability of Human Gastric Cancer Cells

**Figure 2:**
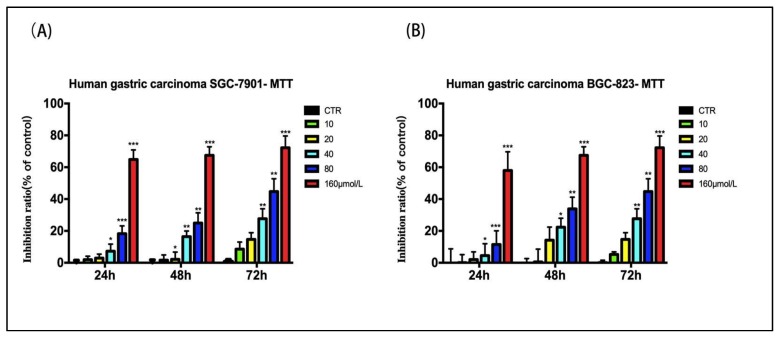
(**A**) Growth inhibitory effects of 28-hydroxy-3-oxoolean-12-en-29-oic acid on SGC-7901. (**B**) Growth inhibitory effects of 28-hydroxy-3-oxoolean-12-en-29-oic acid on BGC-823. Cells were treated with various concentrations of 28-hydroxy-3-oxoolean-12-en-29-oic acid (10, 20, 40, 80 and 160 µmol/L) for 24 h, 48 h and 72 h, and cell viability was assessed by an MTT (thiazole blue) assay.

Control SGC-7901 and BGC-823 cells exhibited active growth in vitro, whereas cells treated with varying concentrations of 28-hydroxy-3-oxoolean-12-en-29-oic acid for 24, 48, or 72 h showed significantly inhibited growth (Figure 2A,B, *p < 0.05, ** *p* < 0.01 and *** *p* < 0.001). The IC50 values of 28-hydroxy-3-oxoolean-12-en-29-oic acid in SGC-7901 and BGC-823 cells were 156.03 μmol/L and 142.2 μmol/L, respectively. Therefore, 28-hydroxy-3-oxoolean-12-en-29-oic acid concentrations of 40, 80, and 160 μmol/L were used in subsequent experiments to exclude the cytotoxic effect of 28-hydroxy-3-oxoolean-12-en-29-oic acid on cell invasion and migration.

### 2.3. 28-Hydroxy-3-oxoolean-12-en-29-oic Acid Prevents the Migration and Invasion of Gastric Cancer Cells

**Figure 3:**
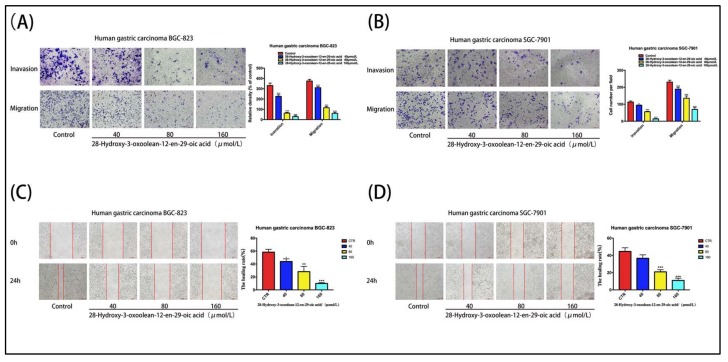
(**A**) Cell invasion assays were conducted using 24-well Transwell chambers with 8.0-μm pore size polycarbonate membranes precoated with Matrigel. After treatment with various concentrations of 28-hydroxy-3-oxoolean-12-en-29-oic acid (40, 80 and 160 μmol/L) for 24 h, the invaded cells on the lower membrane surfaces were fixed and stained with 5% crystal violet solution. (**B**) Cell migration assays did not use Matrigel coating. Images of SGC-7901 and BGC-823 cells were acquired under a microscope at 200× magnification. (**C**,**D**) 28-Hydroxy-3-oxoolean-12-en-29-oic acid treatment for 24 h inhibited the migration of SGC-7901 cells and BGC-823 cells in a wound-healing assay in a dose-dependent manner. Images of SGC-7901 and BGC-823 cells were acquired under a microscope at 200× magnification.

We further examined the effects of 28-hydroxy-3-oxoolean-12-en-29-oic acid on the migration and invasion of gastric cancer cells using Transwell invasion and wound-healing assays. In the Transwell migration and invasion assays, 28-hydroxy-3-oxoolean-12-en-29-oic acid treatment significantly and dose-dependently decreased the number of cells crossing the membrane (Figure 3A,B, * *p* < 0.05, ** *p* < 0.01 and *** *p* < 0.001), indicating that 28-hydroxy-3-oxoolean-12-en-29-oic acid inhibited cell migration and invasion. Furthermore, in the wound-healing assay, 28-hydroxy-3-oxoolean-12-en-29-oic acid treatment for 24 h significantly inhibited the migration of SGC-7901 and BGC-823 cells in a dose-dependent manner (Figure 3C,D, * *p* < 0.05, ** *p* < 0.01 and *** *p* < 0.001).

### 2.4. PerkinElmer Operetta CLS High-Content Imaging System Analysis

**Figure 4:**
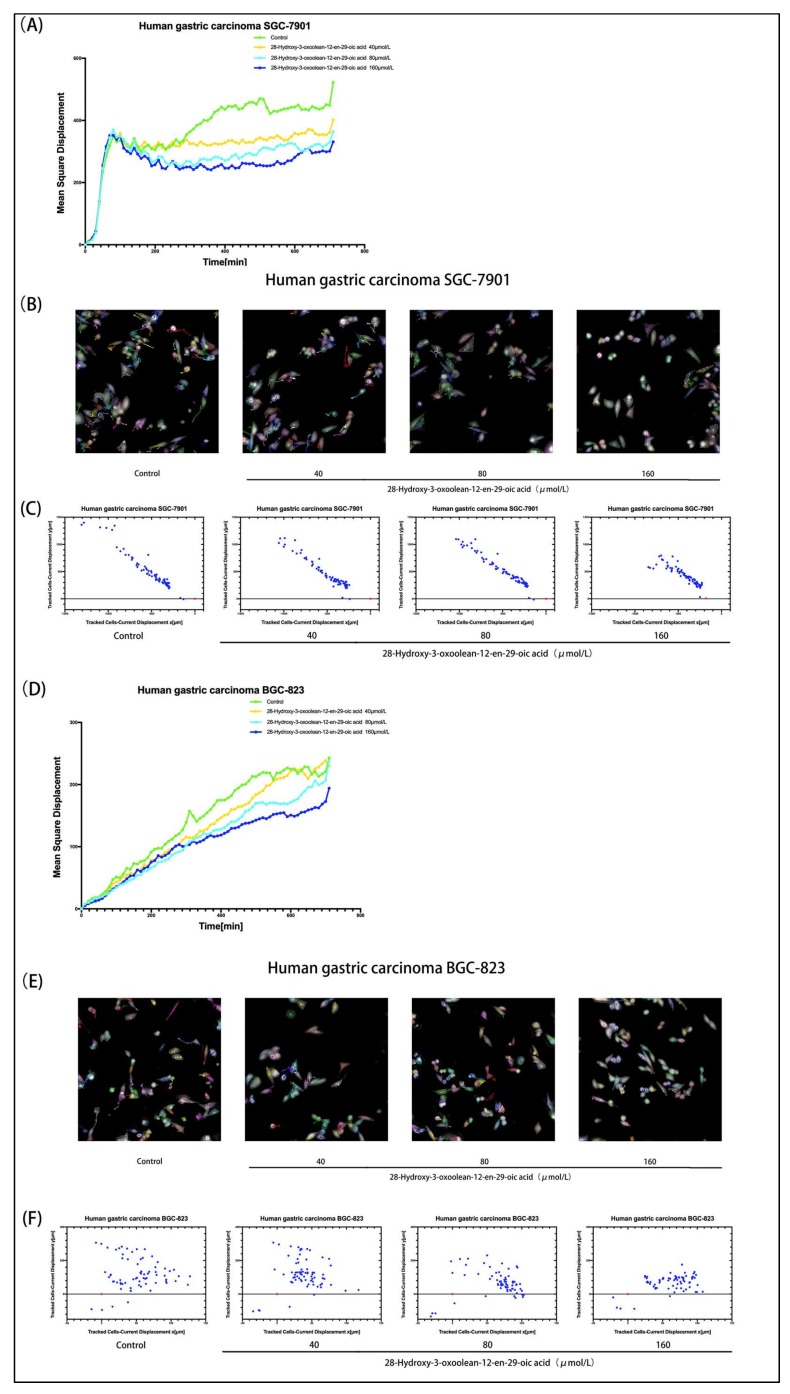
(**A**,**D**) After treatment with various concentrations of 28-hydroxy-3-oxoolean-12-en-29-oic acid (40, 80 and 160 μmol/L) for 24 h, the mean square displacement was plotted vs. the observation time from well-level data. A chemokinesis assay was performed with digital phase contrast (DPC) imaging using single-cell tracking. (**B**,**E**) After treatment with various concentrations of 28-hydroxy-3-oxoolean-12-en-29-oic acid (40, 80 and 160 μmol/L) for 12 h, SGC-7901 cells and BGC-823 cells were seeded and imaged with the 20× objective in the DPC channel. Cells were identified using the Find Cells module, and migration was monitored for 12 h using the Track Objects module. (**C**,**F**) Cell displacement was visualized. Current displacement Y was plotted against current displacement X using the Multiple Graphs module for display. Each point corresponds to the displacement of a cell at a given time point.

We used high-content imaging technology to track the migration of cells more clearly and visually. Analysis with Harmony 4.1 software indicated that the mean square displacement of cells treated with graded concentrations of 28-hydroxy-3-oxoolean-12-en-29-oic acid showed a decreasing trend with increasing observation time (Figure 4A,D). The cells were unlabeled and were imaged in the digital phase contrast (DPC) channel. The DPC image was used to segment the image, and the segmented cells were then tracked. With the increase in drug concentration, little or no cell migration was observed (Figure 4B,E).

### 2.5. Effect of 28-Hydroxy-3-oxoolean-12-en-29-oic Acid on the Expression of Matrix Metalloproteinases (MMPs) in Human Gastric Cancer Cells

**Figure 5:**
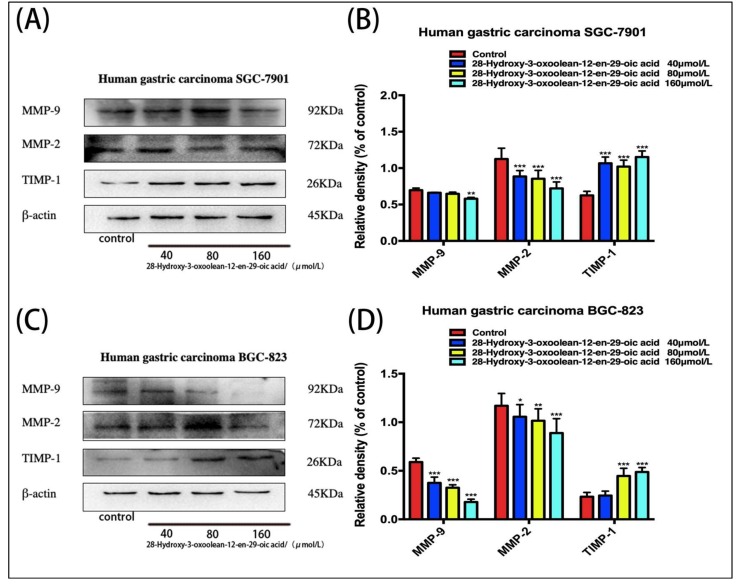
(**A,C**) Changes in the expression levels of matrix metalloproteinases (MMPs) following treatment with 28-hydroxy-3-oxoolean-12-en-29-oic acid for 24 h were assessed by Western blotting. (**B,D**) The band intensities of TIMP-1,MMP-9 and MMP-2 relative to untreated control cells were quantified upon normalizing to β-actin expression, and are expressed as the mean ± standard deviation of three independent experiments.

SGC-7901 and BGC-823 cells treated with 28-hydroxy-3-oxoolean-12-en-29-oic acid for 24 h showed dose-dependent increases in TIMP-1 expression and decreases in MMP-9 and MMP-2 expression (Figure 5A,C, * *p* < 0.05, ** *p* < 0.01 and *** *p* < 0.001).

### 2.6. Effect of 28-Hydroxy-3-oxoolean-12-en-29-oic Acid on Expression of Epithelial–Mesenchymal Transition (EMT)-Related Proteins in Human Gastric Cells

**Figure 6:**
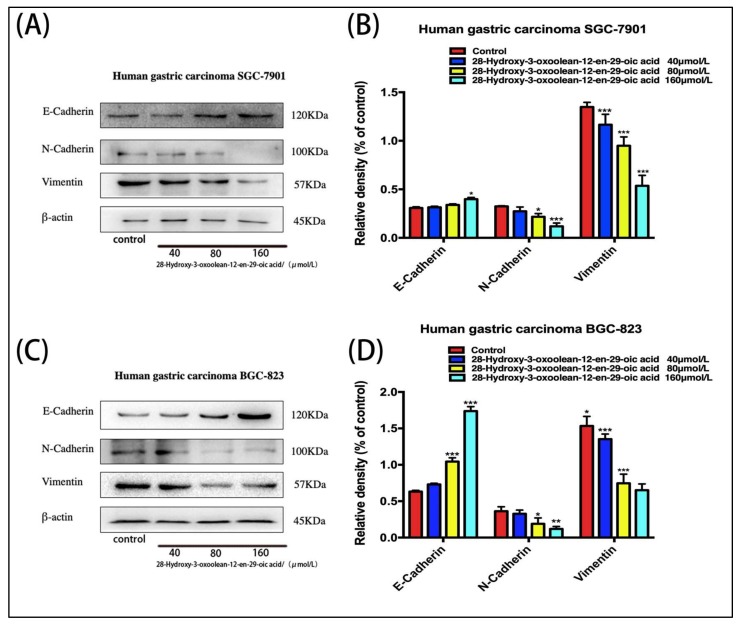
(**A**,**C**) Changes in epithelial–mesenchymal transition (EMT) biomarker expression levels following 28-hydroxy-3-oxoolean-12-en-29-oic acid for 24 h were assessed by Western blotting. (**B**,**D**) The band intensities of E-cadherin, N-cadherin and vimentin relative to untreated control cells were quantified upon normalizing to β-actin expression, and are expressed as the mean ± standard deviation of three independent experiments.

SGC-7901 cells and BGC-823 cells treated with 28-hydroxy-3-oxoolean-12-en-29-oic acid for 24 h showed increased expression of E-cadherin and decreased expression of N-cadherin and vimentin in a dose-dependent manner (Figure 6A,C, * *p* < 0.05, ** *p* < 0.01 and *** *p* < 0.001).

### 2.7. Effect of 28-Hydroxy-3-oxoolean-12-en-29-oic Acid on the Expression of PI3K/Akt/Snail Signaling Proteins in Human Gastric Cancer Cells

**Figure 7:**
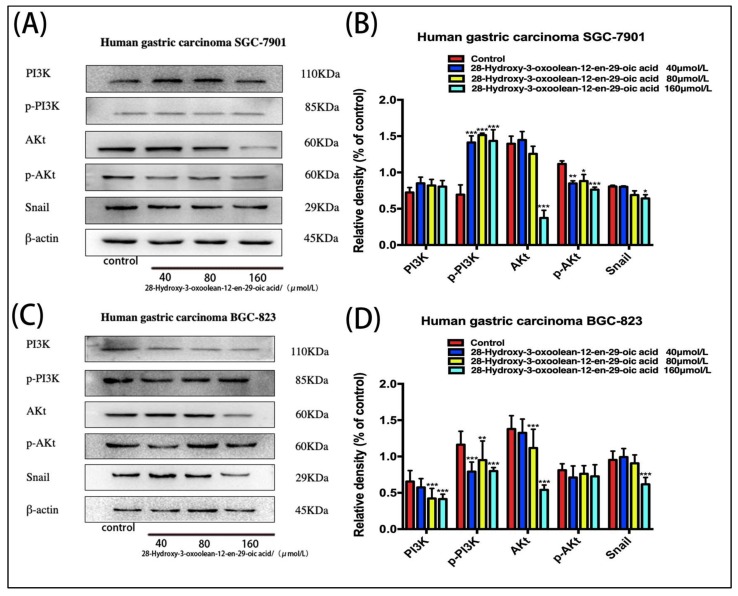
(**A**,**C**) Changes in PI3K/AKt/Snail biomarker expression levels following treatment with 28-hydroxy-3-oxoolean-12-en-29-oic acid for 24 h were assessed by Western blotting. (**B**,**D**) The band intensities of AKt, (p)-Akt, PI3K, (p)-PI3K and Snail relative to untreated control cells were quantified upon normalizing to β-actin expression, and are expressed as the mean ± standard deviation of three independent experiments.

SGC-7901 and BGC-823 cells treated with 28-hydroxy-3-oxoolean-12-en-29-oic acid for 24 h showed dose-dependent decreases in the levels of AKt, (p)-Akt, PI3K, (p)-PI3K and Snail (Figure 7A,C, * *p* < 0.05, ** *p* < 0.01 and *** *p* < 0.001).

## 3. Discussion

According to the existing research, *Celastrus orbiculatus* extract can inhibit the invasion and metastasis of gastric cancer cells [13]. However, whether the individual components the *Celastrus orbiculatus* extract have the same anticancer effect as *Celastrus orbiculatus* extract has not been fully elucidated. This study investigated whether the oleanane-type triterpenoid acid 28-hydroxy-3-oxoolean-12-en-29-oic acid can inhibit the invasion and metastasis of gastric cancer cells and explored its potential molecular mechanism.

The MTT assay results showed that 28-hydroxy-3-oxoolean-12-en-29-oic acid has a concentration-dependent antiproliferative effect on gastric cancer cells. We then investigated whether 28-hydroxy-3-oxoolean-12-en-29-oic acid affects the invasion and metastasis of gastric cancer cells. The results of Transwell and wound-healing assays showed that the invasion and migration ability and wound closure ability of SGC-7901 and BGC-823 gastric cancer cells treated with 28-hydroxy-3-oxoolean-12-en-29-oic acid were significantly reduced. To further clearly observe the invasion and metastasis of gastric cancer cells, we used high-content imaging cell-tracking technology, and these results further confirmed that 28-hydroxy-3-oxoolean-12-en-29-oic acid can inhibit gastric cancer cell migration.

To explore the molecular mechanism by which 28-hydroxy-3-oxoolean-12-en-29-oic acid inhibits the invasion and migration of SGC-7901 and BGC-823 gastric cancer cells, we used Western blotting to measure the protein levels of mmp-2, mmp-9 and timp-1 and found that the expression levels of mmp-9 in 28 hydroxy-3-oxoolean-12-en-29-oic acid-treated sgc-7901 and bgc-823 gastric cancer cells were decreased, while that of timp-1, the inhibitory factor of mmp-2 and mmp-9, was increased. The extracellular matrix plays a very important role in different cells and tissues. It can support and connect tissue structures and can regulate tissue growth and cellular physiological activities [14]. Matrix metalloproteinases (MMPs) are important components of extracellular matrix degradation that can degrade the basement membrane and type IV collagen, a key component of the extracellular matrix—providing a mobile pathway for cancer cell invasion and metastasis [15]. Specifically, among MMPs, MMP-2 and MMP-9 are most closely related to the invasion and metastasis of tumor cells. TIMPs are specific inhibitors of MMPs involved in the control of local activity in tissues, and balance of the extracellular matrix is mainly determined by the close interaction between MMPs and TIMPs. TIMP-1 binds to MMP-9 and MMP-2, thereby inhibiting the functions of these two proteins [16]. Therefore, 28-hydroxy-3-oxoolean-12-en-29-oic acid may inhibit the invasion and migration of gastric cancer cells by downregulating the protein expression of MMP-2 and MMP-9. The mechanism may operate through upregulation of TIMP-1.

To further investigate the molecular mechanism by which 28-hydroxy-3-oxoolean-12-en-29-oic acid inhibits the invasion and metastasis of SGC-7901 and BGC-823 gastric cancer cells, we also examined the levels of E-cadherin, N-cadherin and vimentin. These proteins play different roles in the invasion and migration of gastric cancer cells. E-cadherin mediates the interconnection of allogeneic cells and is a member of a family of calcium-dependent adhesion molecules that mediate cell-to-cell adhesion in order to maintain proper tissue morphology and polarity and participate in intracellular signal transduction [17]. When E-cadherin expression is reduced or absent, intercellular adhesion decreases [18]. Conversely, a decrease in the level of N-cadherin indicates a recovery of cell polarity, thereby inhibiting tumor cell invasion and migration [19]. In addition, a reduction in vimentin levels has also been shown to be associated with tumor cell migration and invasion [20]. EMT is an important biological process by which cancer cells acquire migratory and invasive capabilities. The levels of E-cadherin, N-cadherin and vimentin can be used as indicators of EMT [21]. In this study, we found that the expression of E-cadherin in SGC-7901 and BGC-823 gastric cancer cells treated with 28-hydroxy-3-oxoolean-12-en-29-oic acid was increased, while that of N-cadherin and vimentin was decreased., indicating that 28-hydroxy-3-oxoolean-12-en-29-oic acid may hinder the progression of EMT in gastric cancer cells, thus inhibiting invasion and migration.

Early studies have suggested that Snail plays an important role in the development of the embryonic mesoderm and nervous system through epithelial mesenchymalization. As the important role of epithelialization in tumorigenesis has been confirmed, Snail has gradually become a popular target in cancer research [22]. Snail, as an inhibitor of the E-cadherin gene, is also involved in the invasion and metastasis of gastric cancer cells [23]. AKT is a downstream effector in the PI3K pathway, which plays an important role in regulating tumor cell growth and proliferation, promoting cell invasion and metastasis, and promoting neovascularization [24]. Studies have shown that the PI3K/AKT signaling pathway regulates the expression of the transcription factor Snail, thereby inducing EMT [25]. In this study, we found that the levels of the Snail protein in SGC-7901 and BGC-823 gastric cancer cells treated with 28-hydroxy-3-oxoolean-12-en-29-oic acid were significantly decreased, along with those of AKT, p-AKT, and PI3K. The protein level of p-PI3K also showed a decreasing trend. It is speculated that 28-hydroxy-3-oxoolean-12-en-29-oic acid may regulate the expression of the transcription factor Snail by inhibiting the phosphorylation of AKT/PI3K, thereby blocking EMT in gastric cancer cells.

In summary, this study demonstrates that the oleanane-type triterpenoid acid 28-hydroxy-3-oxoolean-12-en-29-oic acid can inhibit the invasion and migration of gastric cancer cells in vitro. The underlying mechanism may be related to the regulation of EMT via multiple signaling pathways.

This study also showed that the oleanane-type triterpenoid acid 28-hydroxy-3-oxoolean-12-en-29-oic acid has the same in vitro anti-invasive effect in gastric cancer as *Celastrus orbiculatus* extract. Moreover, this study will provide a more solid theoretical and experimental basis for the further studies, including in vivo experiments, necessary for a possible development of *Celastrus orbiculatus* extract components as antitumor drugs. 

## 4. Materials and Methods

### 4.1. Drug

28-Hydroxy-3-oxoolean-12-en-29-oic acid (Yuanye Biotechnology Co., Ltd., Shanghai, China) was dissolved in 1 mmol/L dimethyl sulfoxide (DMSO) before use, and was formulated into a 100 mmol/L mother liquor in serum-free Roswell Park Memorial Institute (RPMI-1640) medium and filtered through a 0.22-μm sterile microporous membrane. For experiments, the solution was diluted with RPMI 1640 medium to the required working concentration, and all working concentrations were cultured. The maximum concentration of DMSO in the base solution did not exceed 0.1%.

### 4.2. Reagents

The reagents used included the following: RPMI 1640 cell culture medium, trypsin (HyClone, USA); fetal bovine serum (FBS) (Gibco, Waltham, MA, USA); artificially reconstituted basement membrane, Transwell chambers (Corning, Corning, NY, USA); thiazole blue (MTT) powder (Sigma, St. Louis, MO, USA); antibodies specific for E-cadherin, N-cadherin, vimentin, Snail, MMP-2, MMP-9, Akt, p-AKt, PI3K, p-PI3K, and β-actin (β-actin) (Cell Signaling, Danvers, MA, USA); and HRP-labeled goat anti-rabbit immunoglobulin (Ig) G (Hangzhou Huaan Biotechnology Co., Ltd., Hangzhou, China). All other chemical reagents used in this study were of analytical grade and obtained from commercial sources.

### 4.3. Cell Culture

The human gastric cancer cell lines SGC-7901 and BGC-823 were obtained from the Cell Bank of the Chinese Academy of Sciences, Shanghai Institute of Cell Biology (Shanghai, China). Cells were cultured in RPMI 1640 medium (HyClone, Thermo Fisher Scientific Biochemical Products Co., Ltd., China) containing 10% FBS and a 1% antibiotic mixture (100 μg/mL streptomycin and 100 U/mL penicillin). Cells were incubated in a humidified atmosphere containing 5% CO_2_ at 37 °C.

### 4.4. Cell Viability Assay

Cells were seeded in a 96-well plate at a density of 6 × 10^3^ cells/well. Subsequently, cells were incubated overnight after treatment with different concentrations of 28-hydroxy-3-oxoolean-12-en-29-oic acid (10, 20, 40, 80 and 160 μmol/L) and further cultured for 24 h, 48 h and 72 h, after which 20 μL of 3-(4,5-dimethyl-2-thiazolyl)-2,5-diphenyl-2-H-tetrazolium bromide (MTT) was added to each well in the dark. After 4 h, 100 μL of DMSO was added after the supernatant was discarded. The absorbance (A) value was read at 490 nm using a microplate reader (Bio-Rad, Nebraska, USA). The experiment was repeated three times. The inhibition rate (%) was calculated as follows: [1—(A of cells in the drug group/A of cells in the control group)] × 100%. The 50% inhibitory concentration (IC50) values were also calculated.

### 4.5. Cell Invasion and Migration Assay

For the wound healing assay, SGC-7901 and BGC-823 cells were cultured in RPMI 1640 medium at a concentration of 5 × 10^5^ cells/mL to 90% confluence. Micropipette tips were used to make linear scratches, and the exfoliated cells were removed by three washes with phosphate-buffered saline (PBS). 3Cells in the 28-hydroxy-3-oxoolean-12-en-29-oic acid groups were treated with 40, 80 or 160 μmol/L and cultured for another 24 h before images were acquired. The experiment was repeated three times. The degree of wound healing (%), calculated as [(scratch width of the control group-scratch width of the 28-hydroxy-3-oxoolean-12-en-29-oic acid group)/scratch width of the control group] × 100%, was used to measure the migration capacity of cells.

Cell invasion assays were performed using 24-well Transwell chambers with 8.0-μm pore size polycarbonate membranes (Corning). After treatment with various concentrations of 28-hydroxy-3-oxoolean-12-en-29-oic acid (40, 80 and 160 μmol/L) for 24 h, cells (5 × 10^4^) were seeded in the upper chambers with membranes precoated with Matrigel (Corning). After incubation, the cancer cells in the upper chamber were removed with cotton swabs. The invaded cells on the lower membrane surface were then fixed and stained with a 5% crystal violet solution. For cell migration assays, Matrigel precoating was not used. Images were acquired under a microscope at 200× magnification (Nikon, Chiyoda-Ku, Tokyo, Japan). Three images in ten random fields of each membrane were acquired, and the migrated cells were counted.

### 4.6. PerkinElmer Operetta CLS High-Content Imaging System Analysis

Cells were seeded in a 96-well plate at a density of 5 × 10^3^ cells/well and subsequently incubated overnight after treatment with different concentrations of 28-hydroxy-3-oxoolean-12-en-29-oic acid (40, 80 and 160 μmol/L) and further cultured for 12 h. The plate was placed into a PerkinElmer Operetta CLS high-content imaging analysis system (PerkinElmer, Waltham, USA) and observed for 12 h. Then, data acquisition and analysis were performed with Harmony 4.1 software.

### 4.7. Western Blot Analysis

SGC-7901 and BGC-823 cells were seeded in a 6-well culture dish, after which they were treated with RPMI 1640 medium containing 28-hydroxy-3-oxoolean-12-en-29-oic acid (40, 80 and 160 μmol/L) for 24 h and cultured for 24 h. Subsequently, cells were lysed with sodium dodecyl sulfate polyacrylamide gel electrophoresis (SDS-PAGE) buffer using standard methods. Protein lysates were separated on a 10% SDS-PAGE gel and transferred to nitrocellulose membranes. Membranes were blocked with 5% skim milk for 2 h at room temperature. Membranes were incubated with primary antibody followed by HRP-conjugated anti-IgG at room temperature for 2 h. Signals were visualized by enhanced chemiluminescence (ECL). A gel imaging analysis system (Bio-Rad) was used to detect the protein bands. The antibodies, including rabbit anti-β-actin, anti-E-cadherin, anti-N-cadherin, anti-vimentin, anti-MMP-2, anti-MMP-9, anti-AKt, anti-phosphorylated (p)-Akt, anti-PI3K, anti-(p)-PI3K, and anti-Snail were purchased from Cell Signaling Technology (Beverly, MA, USA). HRP-labeled goat anti-rabbit IgG was purchased from Hangzhou Huaan Biotechnology Co.

### 4.8. Statistical Analysis

Each experiment was repeated at least three times. Data were analyzed by one-way analysis of variance using SPSS 16.0 (SPSS Inc., Chicago, IL). Data are shown as the means ± standard deviations. *P*-values < 0.05 and *P*-values < 0.01 indicate statistically significant differences.
